# „Ein zu internationaler Berühmtheit gelangter Forscher und Arzt“: Otfrid Foerster (1873–1941) als Nobelpreiskandidat

**DOI:** 10.1007/s00115-021-01184-z

**Published:** 2021-09-15

**Authors:** Lotte Palmen, Ulrike Eisenberg, Axel Karenberg, Heiner Fangerau, Nils Hansson

**Affiliations:** 1 Institut für Geschichte Theorie und Ethik der Medizin, Medizinische Fakultät, Heinrich-Heine-Universität Düsseldorf, Moorenstr. 5, 40225 Düsseldorf, Deutschland; 2 Deutsche Gesellschaft für Neurochirurgie, Berlin, Deutschland; 3 Institut für Geschichte und Ethik der Medizin, Universitätsklinikum Köln, Universität zu Köln, Köln, Deutschland

**Keywords:** Neurologie, Neurochirurgie, Exzellenz, Preise, Deutschland, Neurology, Neurosurgery, Excellence, Awards, Germany

## Abstract

Dieser Aufsatz befasst sich mit den Nobelpreisnominierungen für den Neurologen und Neurochirurgen Otfrid Foerster (1873–1941). Foerster wurde 17 Mal für den Nobelpreis für Physiologie oder Medizin nominiert. Aufbauend auf Akten des Stockholmer Nobelpreisarchives, Primär- und Sekundärliteratur wird auf folgende Fragen eingegangen: Welche Gründe gab es für Foersters Nominierungen? Wie sah die Beziehung zwischen ihm und seinen Nominatoren aus? Warum hat er letztlich den Nobelpreis nicht erhalten? Das Gros der Nominatoren für Foerster hob als Hauptmotiv sein gemeinsam mit Oswald Bumke herausgegebenes *Handbuch der Neurologie* hervor. Den Nominatoren zufolge hatte Foerster mit diesem Handbuch einen enormen Einfluss auf die Neurologie seiner Zeit. Darüber hinaus wurde sein „ehrenvoller Charakter“ in den Nominierungsbriefen unterstrichen. Für das Nobelkomitee waren diese Begründungen jedoch nicht ausreichend: Die Mitglieder stuften das Handbuch nicht als originäre Forschungsleistung ein. Foersters Ruhm reicht trotzdem bis in die Gegenwart, etwa in Form einer seit 1953 von der Deutschen Gesellschaft für Neurochirurgie vergebenen Ehrung, die seinen Namen trägt (Otfrid-Foerster-Medaille).

## Abstract (en)

This paper discusses the 17 Nobel Prize nominations for the neurologist and neurosurgeon Otfrid Foerster (1873–1941). Drawing on files from the Stockholm Nobel Prize Archive, primary and secondary literature, it addresses the following questions: what were the reasons given by nominators for Foerster’s nominations? What was the relationship between him and his nominators? Why was he ultimately not awarded the Nobel Prize? Most nominators of Foerster’s highlighted as the main motive his *Handbuch der Neurologie*, which he had edited with Oswald Bumke. According to the nominators, this book together with Foerster’s neurosurgical work had an enormous impact on contemporary neurology. Furthermore, his “honorable character” was underlined in the nomination letters; however, these reasons were not sufficient for the Nobel Committee: the members classified the handbook as not being original research. Despite this, Foerster’s fame is reflected in the present, for example in the Otfrid Foerster Medal, which has been awarded to researchers by the German Society of Neurosurgery since 1953.

## Hintergrund

Seitdem der Nobelpreis 1901 erstmals vergeben wurde, gilt er als höchste Auszeichnung, mit der Pioniere in den Gebieten Medizin, Chemie, Physik, Literatur und Friedensbemühungen gekürt werden, wenn ihre Leistungen dem „größten Nutzen für die Menschheit“ [16] dienen. Ein Forschungsprojekt an der Heinrich-Heine-Universität Düsseldorf befasst sich mit der Geschichte des Nobelpreises, um verschiedene Definitionen von Exzellenz in den Wissenschaften zu analysieren. Der Preis und seine Vergabeprozesse werden dabei als stellvertretend für Ergebnisse und Abläufe der Aushandlung von wissenschaftlicher Exzellenz begriffen. Es geht also nicht nur um die Laureaten, die am Ende tatsächlich geehrt werden, sondern auch um die vergeblich von ihren Kollegen für den Preis vorgeschlagene Kandidaten. Nobelpreisnominierungen sind ideale Quellen, um mehr über den Ruf einzelner Forscher und aktuelle Forschungstrends zu einem bestimmten Zeitpunkt zu erfahren. Eine jüngst erschienene Übersicht über die zwischen 1901 und 1950 nominierten Neurologen aus fünf europäischen Ländern (Deutschland, Frankreich, Großbritannien, Italien und Russland) benannte Gründe, warum einige der nominierten Neurologen zwar große Forschungserfolge erzielten und sich dadurch weltweit einen Namen machten, wie etwa Eduard Hitzig (1838–1907) und Joseph Babinski (1857–1932), den Nobelpreis aber am Ende *nicht* erhielten [9]. So argumentierten Mitglieder der Preisjury beispielsweise, dass Hitzigs Arbeiten über die Lokalisationslehre „zu alt“ und Babinskis Forschungsinteressen zu disparat gewesen seien. Darüber hinaus sind auch die Neurologen Heinrich Quincke (1842–1922), Wilhelm Erb (1840–1921), Paul Flechsig (1847–1929), Franz Nissl (1860–1919), Cécile Vogt (1875–1962), Oskar Vogt (1870–1959) und Hans Berger (1873–1941) für den Preis nominiert worden (Tab. 1).

**Table 1:** 

Jahr der Nominierung	Kandidat	Nominatoren	Gründe
1940–1950, 6 Nominierungen	Hans Berger	Lord E. Adrian	Entwicklung der Elektroenzephalographie
		W. Cannon	
		T. Putnam	
		L. Benedek	
		P. Redaelli	
		C. Pfeiffer	
1910, 3 Nominierungen	Ludwig Edinger	K. Heilbronner	Forschung zur Hirnentwicklung
		C. Spronck	
		H. Lameris	
1910–1914, 2 Nominierungen	Wilhelm Erb	N. Ortner	Arbeiten zur Elektrodiagnostik und Neuropathologie
		H. Ortner	
1923–1924, 2 Nominierungen	Paul Flechsig	H. Held	Forschung an neuronalen Leitungswegen des Gehirns und Rückenmarks
		H. Curschmann	
1926–1937, 17 Nominierungen	Otfrid Foerster	W. Weygandt	Klinische Forschung in der Neurologie, vor allem Epilepsieforschung, Arbeiten über die Anatomie und Physiologie des peripheren Nervensystems, sein Werk „Symptomatologie des Rückenmarks und seiner Wurzeln“ (1936)
		B. Pfeifer	
		E. Friedmann	
		L. Fraenkel	
		H. Curschmann	
		R. Perwitzschky	
		R. Wagner	
		B. Dürken	
		M. Staemmler	
		J. Lange	
		E. Schmitz	
		H. Euler	
		F. Schultze-Rohnhof	
		K. Gutzeit	
		O. Eichler	
		K. Stolte	
1904–1906, 5 Nominierungen	Eduard Hitzig	A. von la Valette-St. George	Lokalisationslehre, seine Arbeit „Physiologische und Klinische Untersuchungen über das Gehirn“ (1904)
		H. Fischer	
		H. Eichhorst	
		E. Siemerling	
		A. Lucae	
1914–1919, 2 Nominierungen	Franz Nissl	P. Ernst	Forschung am Nervensystem und zur Heilung von Nervenkrankheiten
		E. Kraepelin	
1919, 1 Nominierungen	Hermann Oppenheim	Nathan Zuntz	Forschungen zur Diagnose und Therapie von Nervenkrankheiten
1909–1922, 17 Nominierungen	Heinrich Quincke	F. Hoffmann	Forschung zur Lumbalpunktion
		A. Bier	
		B. Naunyn	
		G. Hoppe-Seyler	
		C. Binz	
		P. Krause	
		W. Kruse	
		C. Pelman	
		F. Schultze	
		R. Bonnet	
		H. Leo	
		L. Merk	
		F. Hoffmann	
		I. Holmgren	
		L. Müller	
		R. Stintzing	
1920–1950, 11 Nominierungen	Oskar Vogt	H. Liepmann	Forschungen zur Architektur der Großhirnrinde, zur Physiologie und Pathologie des Gehirns, zur Alterung von Gliazellen und ihrer Veränderung bei Schizophrenie
		R. Bárány	
		G. Bergmark	
		E. Holmgren	
		K. Kleist	
		W. Weygandt	
		E. Forster	
		A. Policard	
		O. Foerster	
		F. Volhard	
1922–1950, 9 Nominierungen	Cécile Vogt	R. Bárány	Forschungen zur Architektur der Großhirnrinde, zur Physiologie und Pathologie des Gehirns, zur Alterung von Gliazellen und ihrer Veränderung bei Schizophrenie
		G. Bergmark	
		E. Holmgren	
		K. Kleist	
		W. Weygandt	
		E. Forster	
		Policard	
		F. Volhard	
1926–1932, 3 Nominierungen	Constantin v. Economo	A. Kousis	Forschung zur Encephalitis lethargica
		H. Marcus	
		F. von Müller	

Die Relevanz neurologischer Themen im Nobelpreiskontext zeichnete sich schon im ersten Nobelpreisjahrzehnt ab: 1906 ging der erste für neurowissenschaftliche Forschungen vergebene Nobelpreis für Neurologie an Camillo Golgi (1843–1926) und Santiago Ramon y Cajal (1852–1934) für ihre Arbeiten zur Struktur des Nervensystems. In den darauffolgenden Dekaden traten immer öfter auch Neurochirurgen in den Vordergrund, auf internationaler Basis vor allem Harvey Cushing (1869–1939), der insgesamt 38 Mal zwischen 1917 und 1939 nominiert wurde [8]. Bei den Neurowissenschaftlern aus Deutschland stechen insbesondere Otfrid Foerster und Heinrich Quincke heraus: Beide wurden 17 Mal für den Nobelpreis nominiert. Während über Quincke als Nobelpreiskandidat – er wurde dabei v. a. als Erfinder der Lumbalpunktion inszeniert – bereits eine Publikation vorliegt [6], gibt es bisher keine detaillierte Studie über die Nobelpreisnominierungen für Otfrid Foerster. Warum wurde er nominiert und weshalb hat er den Nobelpreis nicht erhalten?

## Biografische Skizze und Forschungsstand

Otfrid Foerster wurde am 09.11.1873 in Breslau geboren, studierte von 1892 bis 1896 an den Universitäten Freiburg, Kiel und Breslau Medizin und wurde 1897 in seiner Heimatstadt promoviert. Seine fachärztliche Weiterbildung in der Neurologie absolvierte er unter anderem bei Carl Wernicke (1848–1905). Wernickes neuroanatomische Arbeiten (er entdeckte 1874 die nach ihm benannte sensorische Sprachregion) prägten Foersters Bestrebungen, Neuroanatomie, Neurophysiologie und klinische Neuropathologie zu verbinden. Seine Forschungen zum Rückenmark führten ihn ab 1908 dazu, in diesem Bereich auch operativ tätig zu werden. Zu Beginn führte er spinale Operationen zusammen mit den Breslauer Chirurgen Alexander Tietze (1864–1927), Chef im Allerheiligen-Hospital, und Hermann Küttner (1870–1932), Ordinarius für Chirurgie, durch. Er entwickelte die „dorsale Rhizotomie“, um die spastische Tonussteigerung bei Schädigungen der Rückenmarkswurzeln zu mindern – diese Methode ist seither nach ihm benannt [22]. Im weiteren Verlauf operierte er verletzte periphere Nerven, Rückenmarksgeschwülste und nach Ende des Ersten Weltkriegs zunehmend auch Hirntumoren, immer in Lokalanästhesie.

Foerster habilitierte sich 1903 für Neurologie und Psychiatrie mit einer Arbeit über Koordinationsstörungen [1]. Zusammen mit Wernicke veröffentlichte er in jenem Jahr auch einen fotografischen „Atlas des Gehirns“ [21]. Auf Wernickes Vorschlag verbrachte Foerster zwei Jahre im Ausland, in Paris und in der Schweiz [22]. 1911 wurde er leitender Arzt der Nervenabteilung des Allerheiligen-Hospitals, 1917 erhielt er dort als Honorarprofessor ein persönliches, auf ihn zugeschnittenes Ordinariat für Neurologie, eines der ersten in Deutschland. Ab 1921 war er dann als ordentlicher Professor der Neurologie an der Universität Breslau tätig und zwischen 1934 und 1938 Direktor des mithilfe der Rockefeller-Stiftung neu erbauten Neurologischen Forschungsinstitutes Breslau – ein Beweis für seine internationale Reputation.

Foersters Ergobiografie wurde bereits mehrfach thematisiert [12, 19]. So hob der deutsche Psychiater und Neurologe Robert Gaupp (1870–1953) zwei Jahre nach Foersters Tod in dem Artikel „Otfrid Foerster. Sein Lebenswerk im Rahmen der Wissenschaft seiner Zeit“ die Relevanz seiner Forschung für die Neurowissenschaften hervor [4]. Er thematisierte dabei Foersters Werdegang sowie persönliche Beziehungen und gab einen ausführlichen Überblick über Foersters Ausbildung und Forschung. Der Neurowissenschaftler Klaus Joachim Zülch (1910–1988), ehemaliger Schüler von Foerster, gab 1966 zum 25. Todestag Foersters ein Werk heraus, um „das Bild seines Lebens und sein gewaltiges wissenschaftliches Werk in seinen Höhepunkten“ [22] wiederzugeben. Darin wird Foerster inszeniert als „ein Meister der Physiologie und Pathophysiologie des Nervensystems“, der „wie von einem Dämon besessen […] sein fast übermenschlich großes Werk“ [22] erschuf. Zülch gliederte Foersters Forschung in drei Abschnitte: Beiträge zur neurologischen Semiologie, Diagnostik und Klinik, zur Neurochirurgie und zur angewandten Physiologie des Nervensystems. Er gab dadurch unter anderem einen Einblick in Foersters Arbeiten über die Übungstherapie in der Orthopädie, Querschnittssyndrome des Rückenmarks, das Schmerzgefühl und seine Leitungsbahnen, das Lokalisationsprinzip und die sog. Foerstersche Operation.

Foersters Reputation wurde dadurch unterstrichen, dass er ab 1925 erster Vorsitzender der Gesellschaft Deutscher Nervenärzte war und eine Vielzahl von Auszeichnungen erhielt, darunter 1935 während des 2. Internationalen Neurologenkongress in London die goldene Jackson-Gedächtnismedaille. Diese, im Gedenken an den britischen Neurologen John Hughlings Jackson (1835–1911) verliehen, war Foersters höchste Auszeichnung. Der mehrere Sprachen fließend sprechende Foerster war zweifelsohne international bekannt und in den angelsächsischen Ländern gut vernetzt [18]. Regelmäßig besuchten ihn britische und amerikanische Neuroforscher, da es bei „jungen amerikanischen Neurologen und Neurochirurgen zu einer guten Ausbildung gehörte, bei Otfrid Foerster gewesen zu sein“, so retrospektiv zumindest sein Hagiograph Klaus Zülch [22]. Er nahm eine Ausnahmestellung innerhalb der Neurologie ein, die dazu führte, dass er auf dem I. Internationalen Neurologenkongress in Bern 1931 eine von ihm selbst verfasste „Resolution an die Regierung der Weltstaaten“ verlas, in der er für die volle Anerkennung der Neurologie als eigenes akademisches Fach plädierte [22]. Auf diese Resolution und ihre Bedeutung für die Neurologie nahm beispielsweise der damalige zweite Vorsitzende der Gesellschaft Deutscher Neurologen und Psychiater Heinrich Pette (1887–1964) in einer Grabrede auf Foerster zu dessen Beisetzung 1941 Bezug [17]. Seine Kontakte beschränkten sich aber nicht nur auf Europa und Nordamerika: Beispielsweise hospitierte auch Dr. Abdülkadir Cahit Tuner (1892–1980), einer der ersten Neurochirurgen der Türkei, bei Foerster in Breslau, um die Techniken der Neurochirurgie zu erlernen [20].

Ferner wurde Otfrid Foersters Haltung als deutscher Neurologe gegenüber dem Nationalsozialismus analysiert [13]. Aktuellen Forschungen zufolge erkannte das NS-Regime seine Leistungen zwar an, jedoch zählte Foerster, der nie Mitglied der NSDAP war, weder zu den bekannten Unterstützern noch zu den offiziellen NS-Kritikern: „Foerster selbst verstand sich als unpolitischer Wissenschaftler“ [13]. Er hatte eine jüdische Ehefrau und zahlreiche jüdische Assistenten, wie etwa seinen leitenden Oberarzt Ludwig Guttmann (1899–1980). Gleichwohl wurden in seinem Institut mutmaßlich auch Gehirne von Kindern untersucht, die im Rahmen der NS-Kindereuthanasie ermordet worden waren [14]. Ob ihm die Zusammenhänge bekannt waren, bleibt unklar.

## Methode

Grundlage des Aufsatzes sind die Daten des Nobelpreisarchives (nobelprize.org), in welchem einige Angaben über Otfrid Foersters Nominierungen zusammengetragen sind. Darüber hinaus hat der Autor NH Foerster-Akten (Nominierungen und Gutachten) im Archiv des Nobelkomitees für Physiologie oder Medizin in Stockholm eingesehen. Die Nominierungen wurden von der Erstautorin LP transkribiert und anhand der angeführten Primär- und Sekundärliteratur von allen Autoren/-innen kontextualisiert. Die Gutachten des Nobelkomitees wurden aus dem Schwedischen ins Deutsche übersetzt.

## Ergebnis

**Figure 1:**
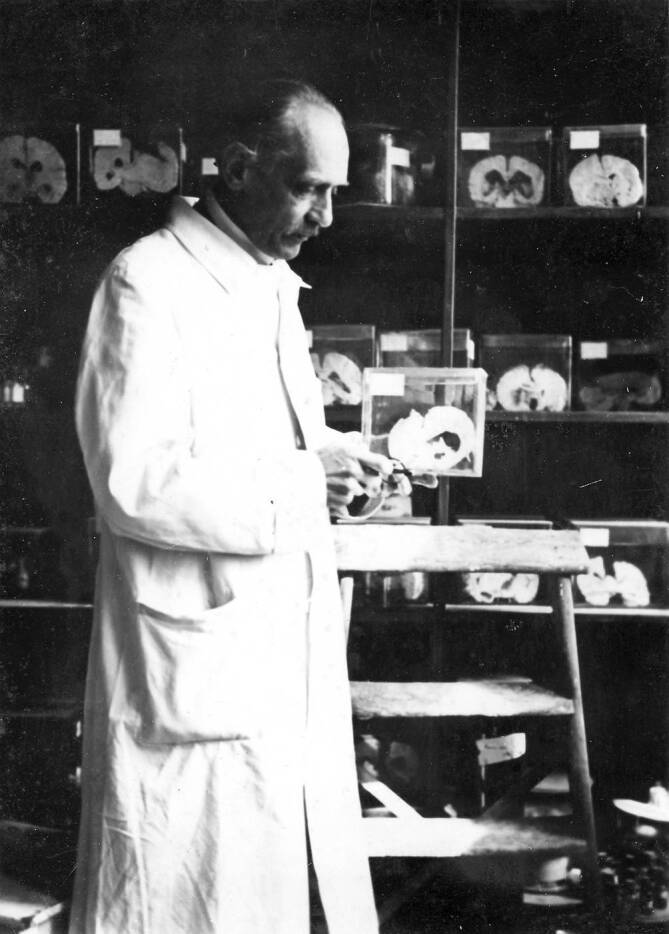

Otfrid Foerster wurde insgesamt 17 Mal für den Nobelpreis nominiert. Allgemeine Themen in den Nominierungen waren die Behandlung von Schmerzen, die Topik des peripheren Nervensystems, Querschnittssyndrome und Epilepsieforschung. Als spezifische Leistungen im Bereich der Neurochirurgie erwähnten die Nominatoren primär Foersters Forschung in den Jahren 1929 bis 1937, die sich in seinen Publikationen „Die Leitungsbahnen des Schmerzgefühles und die chirurgische Behandlung der Schmerzzustände“ (1927; [2]) und „Symptomatologie der Erkrankungen des Rückenmarks und seiner Wurzeln“ (1936; [3]) abbildete. Neben seinen Forschungsergebnissen wurden sein gesamtes Lebenswerk und seine von den Nominatoren geschätzte „Forscherpersönlichkeit“ als unterstützende Nominierungsgründe genannt. Er galt als ein „weit über den Rahmen seines Heimatlandes hinaus gewachsener zur internationalen Berühmtheit gelangter Forscher und Arzt“ [10] und als „Begründer der modernen Neurochirurgie“ [10]. Die Nominatoren, zu denen seine universitären Kollegen Hans Curschmann (1875–1950, Professor für Innere Medizin in Rostock), Reinhard Perwitzschky (1896–1971, Professor für Otorhinolaryngologie in Breslau), Richard Wagner (1893–1970, Professor für Physiologe in Breslau), Bernhard Dürken (1881–1944, Professor für Zoologie in Breslau), Martin Staemmler (1890–1974, Professor für Pathologie in Breslau) und Johannes Lange (1891–1938, Professor für Psychiatrie in Breslau) gehörten, stimmten diesem Tenor mit ihren Nominierungen zu. Unter den Nominatoren finden sich mehrere Kollegen aus Breslau, da die schlesische Metropole im Jahre 1937 ein Vorschlagsrecht hatte. Das Nominierungsverfahren folgte zu dieser Zeit einem bestimmten Schema: Das Lehrkollegium am Karolinska Institut wählte ein „Nobelkomitee“ aus fünf Professoren, die aus allen Nominierungen einen bis drei preiswürdige Kandidaten auszuwählen und dann dem Lehrkollegium für die endgültige Entscheidung vorzuschlagen hatten ([7]; Abb. 1 und 2).

**Figure 2:**
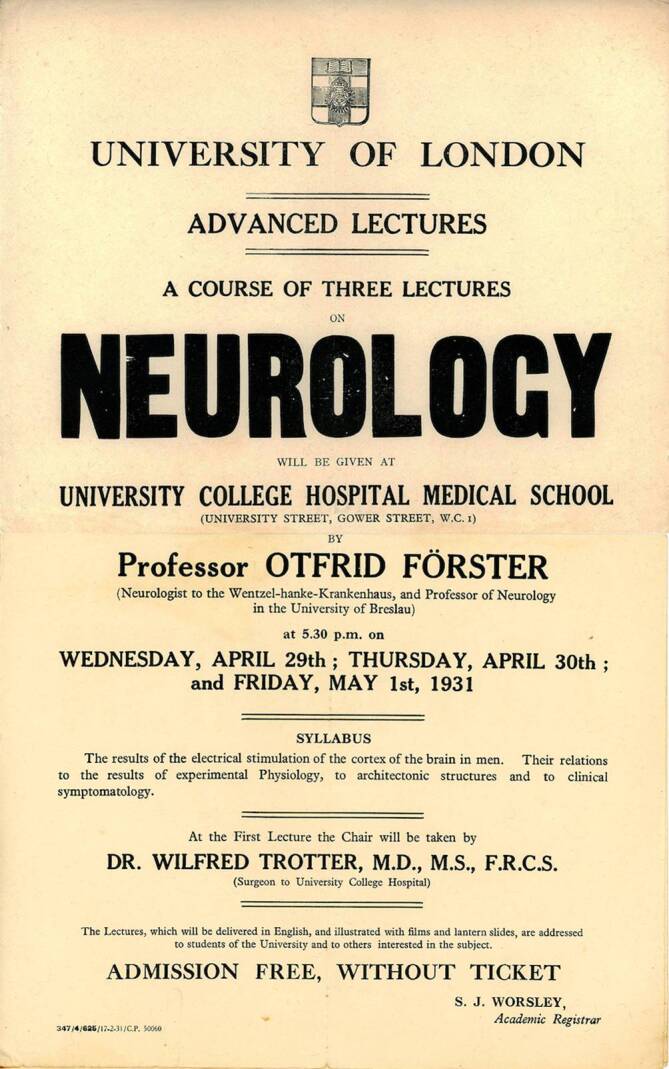

Reinhard Perwitzschky nannte Foerster in seinem Schreiben einen der „größten deutschen Ärzte“ [10], dessen Entdeckungen dazu beigetragen hätten, dass er zu einem Wohltäter der Menschheit geworden sei. R. Wagner, ab 1937 Rektor der Universität, gab eine ähnliche Stellungnahme ab mit den Worten: „Seine Arbeiten haben nicht nur einen beträchtlichen Wert für die klinische Medizin und für die lebende Menschheit, sondern bereichern darüber hinausgehend in entscheidender Weise unsere Kenntnisse über die Funktion des Zentralnervensystems“ [10]; ferner lobte er die „hervorragende Beobachtungsgabe O. Foersters, [und] seine seltene Fähigkeit, das Gesehene einzuordnen“ [10]. Der Psychiater und Neurologe J. Lange, als Breslauer Kollege mit Foersters Forschungen vertraut, nannte Foerster eine „eigenartige Forscherpersönlichkeit, die freilich das eigene Verdienst eher verbirgt als herausstellt“ [10], was seinen ehrenvollen Charakter belege und die geschätzte Position Foersters unter seinen Kollegen widerspiegele. Lange brachte die Leistung Foersters durch seine Aussage „Die Physiologie ist bei Foerster wirklich Helferin der Therapie, jeder seiner therapeutischen Eingriffe aber eine Befruchtung der Physiologie“ [10] auf den Punkt. R. Wagner schrieb weiterhin: „Man muss die Arbeiten Foersters in ihrer Gesamtheit bewerten und beurteilen, um die Erkenntnis zu gewinnen, dass sein Lebenswerk diese hohe wissenschaftliche Auszeichnung rechtfertigt“ [10], was verstärkt, dass die Gesamtleistung Foersters für die Forschung als nobelpreiswürdig erachtet wurde. Foerster wurde demnach eine Art Pionierstellung in den Neurowissenschaften eingeräumt, den Höhepunkt seiner Forschung habe er jedoch erst mit seinem Kapitel „Symptomatologie der Erkrankungen des Rückenmarks und seiner Wurzeln“ im *Handbuch der Neurologie* erreicht.

Als im Jahr 1937 die Mehrzahl seiner insgesamt erzielten Nominierungen eingereicht wurde, stand genau dieses Standardwerk im Fokus. Foerster veröffentlichte das Handbuch in 17 Bänden zusammen mit Oswald Bumke (1877–1950), dem Münchner Ordinarius für Psychiatrie [11]. Es fällt auf, dass vor allem Foersters Eingriffe an Menschen und nicht nur die bisher bekannte Forschung durch Tierversuche die Zuverlässigkeit seiner Arbeit belegten. Seine Beobachtungen bei chirurgischen Eingriffen lieferten Informationen über Aufbau und Funktion des Rückenmarks, er erforschte detailliert die Dermatome, die Schmerzentstehung, die Entstehung von Reflexen. Neu war dabei für die Zeitgenossen, so Bernhard Dürken, die „von physiologisch-morphologischen Forschungen ausgehende Förderung der Therapie“ [10]. Die Nominatoren hatten eine einheitliche hohe Meinung von der Arbeit, die Foersters Lebenswerk in ihren Worten „krönte“ und eine Art Zusammenfassung seiner erreichten Leistungen darstellen sollte. Das Werk zählte als „ein klassisches Werk der Physiologie“ [10] und Foerster selbst galt dabei als „ordnender Geist (der) unsere physiologischen Kenntnisse über das Zentralnervensystem auf Grund sorgfältiger Beobachtungen“ [10] geprägt habe. Foerster wurde so „zu den bahnbrechenden Gestalten der Neurochirurgie“ stilisiert [10].

Eine weitere Begründung für Foersters Nominierungen war seine internationale Reputation weltweit, die sich unter anderem darin widergespiegelte, dass er im Jahre 1922 Wladimir I. Lenin (1870–1924) behandelte und fast anderthalb Jahre mit nur kurzen Unterbrechungen bei ihm blieb. Er hob sich vor allem durch die lange Zeit, die er in Russland verbrachte, von den anderen internationalen Mitgliedern des Ärzteteams an Lenins Krankenbett ab. In einer Nominierung im Jahr 1930 heißt es dazu: „Ich bitte den Umstand, dass der hier Unterzeichnete Breslauer Forscher einen Landsmann vorschlägt, nicht misszuverstehen bzw., auf lokale Einflüsse zu beziehen, denn Otfrid Foerster ist ein weit über den Rahmen seines Heimatlandes hinaus gewachsener zur internationalen Berühmtheit gelangter Forscher und Arzt geworden, den Kranke aus allen Ländern der Welt aufsuchen und der deshalb auch von der russischen Regierung bei der schweren Erkrankung Lenin’s [sic] auf viele Monate nach dort berufen worden ist“ [10].

Den Nominatoren zufolge galt Otfrid Foerster also als ein exzellenter Neurowissenschaftler. Er habe nicht nur die theoretische Medizin weiterentwickelt, sondern vielmehr die praktische Medizin und habe damit der ganzen Menschheit genutzt. Warum ist er dann am Nobelkomitee gescheitert?

## Gutachten

Für Otfrid Foerster wurden zwei Gutachten erstellt, in denen die Aktualität und Relevanz seiner Forschungsergebnisse analysiert wurden. Im Jahre 1926, zum Zeitpunkt von Foersters erster Nominierung, ging der schwedische Neurologe Henry Marcus (1866–1944), nach Studienaufenthalten bei Alois Alzheimer (1864–1915), Emil Kraepelin (1856–1926) und Franz Nissl (1860–1919) in Deutschland gut vernetzt, auf Foersters Epilepsieforschung ein, welche er als noch nicht „reif“ genug für eine ernsthafte Preisdiskussion einstufte. Jedoch konnte sich Marcus gut vorstellen, dass er „künftig durchaus als Preisträger infrage kommt“. 1937 erfolgte eine erneute Stellungnahme, diesmal von dem Stockholmer Neurologen Nils Antoni (1887–1968) verfasst. Wie Marcus verfügte auch Antoni dank mehrerer Studienreisen (etwa Köln-Lindenthal 1917 und Hamburg-Eppendorf 1925) über einen guten Überblick zur neurologischen Forschung in Deutschland. Antoni bezog sich gezielt auf Foersters Hauptnominierungsgrund, das Werk „Symptomatologie der Erkrankungen des Rückenmarks und seiner Wurzeln“, mit der Fragestellung, ob das Handbuch von Foerster und Bumke preiswürdige Entdeckungen liefere. Er unterstrich hierbei einleitend, wie heikel es sei, in einem Handbuchbeitrag originelle und innovative Forschung zu identifizieren, die eindeutig auf Foerster zurückzuführen sei; des Weiteren kam laut Antoni erschwerend dazu, dass „Foerster auf ausführliche Literaturangaben verzichtet“. Nichtsdestotrotz betonte er, dass Foersters Forschungen am Menschen viele neue Ergebnisse geliefert habe [7]. In Antonis Gutachten wurden sechs Forschungsschwerpunkte Foersters erläutert:

1. Die graue Substanz und ihre Verbindungen,
2. Funktion der Hinterstränge,
3. Ursprung der sympathischen Innervation verschiedener Zielorgane,
4. Beiträge zur Physiologie der Seitenstränge,
5. Funktion von Hinter- und Vorderwurzel,
6. Segmentlehre.

Antoni unterstrich zum Schluss, dass einerseits die Entdeckung der zeitdiskriminatorischen Funktion der Hinterstränge und andererseits die Entdeckung spezifischer Vasodilatatoren in den dorsalen Nervenwurzeln für die Forschung an der spinalen Segmentlehre für das Komitee interessant sein könnten.

## Diskussion

Der Begriff Exzellenz hat keine klare Definition, sondern ist vielmehr ein Wert, der einem Menschen von einem anderen zugeschrieben wird. Einfluss auf diese Zuordnung haben subjektive Werte und persönliche Interessen. In Otfrids Foersters Nobelpreisnominierungen wird vorweggenommen, was auch rezentere Publikationen über Foerster immer wieder anführen, nämlich, dass seine Arbeiten weltweit anerkannt seien [4] und er Mitbegründer der Neurochirurgie sei [22]. Auch zähle er noch zu den wichtigsten Neurowissenschaftlern, die Deutschland je hatte, er sei sogar eine der kraftvollsten Persönlichkeiten in der gesamten Medizin [5]. Dies wurde primär durch seine Publikationen begründet, aber auch durch seine vielen Auszeichnungen, etwa den Ehrenvorsitz in der Gesellschaft deutscher Nervenärzte sowie die Mitgliedschaften in der Leopoldina oder der American Neurological Association. Des Weiteren besuchten Neurowissenschaftler aus der ganzen Welt seine Klinik.

Letztlich ging der Preis 1937 an den Biochemiker Albert von Szent-Györgyi (1893–1986) „for his discoveries in connection with the biological combustion processes, with special reference to vitamin C and the catalysis of fumaric acid“. Wir können somit die Vermutung aufstellen, dass die durch Antoni genannten Kritikpunkte in Bezug auf die Schwierigkeit der genauen Zuordnung von Forschungsergebnissen und die zu geringe Spezifität in Foersters Werk der Grund dafür war, dass Foerster im Jahr 1937 trotz mehrfacher Nominierungen und neuer Ergebnisse den Nobelpreis nicht erhalten hat.

## Fazit für die Praxis

Bis heute zählt Otfrid Foerster zu den prägendsten Persönlichkeiten in den Neurowissenschaften und zu einem Pionier der Neurochirurgie, nicht zuletzt durch die Eröffnung eines Neurologischen Forschungsinstituts, das später nach ihm benannt wurde. Die Deutsche Gesellschaft für Neurochirurgie gedenkt seiner noch heute mit Vergabe der Otfrid-Foerster-Medaille. Sie wurde 1953 zum ersten Mal Percival Bailey (1892–1973) überreicht. Darüber hinaus vergibt seit 2004 die Deutsche Gesellschaft für Epileptologie ebenfalls eine Otfrid-Foerster-Medaille sowie ein Otfrid-Foerster-Stipendium. Außerdem gibt es an der Medical School Hamburg die „Otfrid Foerster Lecture“. Somit wird – auch ohne Nobelpreis – durch mehrere Initiativen an Foerster erinnert.
